# Genetics of Proteasome Diseases

**DOI:** 10.1155/2013/637629

**Published:** 2013-12-30

**Authors:** Aldrin V. Gomes

**Affiliations:** ^1^Department of Neurobiology, Physiology, and Behavior, University of California, Davis, CA 95616, USA; ^2^Department of Physiology and Membrane Biology, University of California, Davis, CA 95616, USA

## Abstract

The proteasome is a large, multiple subunit complex that is capable of degrading most intracellular proteins. Polymorphisms in proteasome subunits are associated with cardiovascular diseases, diabetes, neurological diseases, and cancer. One polymorphism in the proteasome gene *PSMA6* (−8C/G) is associated with three different diseases: type 2 diabetes, myocardial infarction, and coronary artery disease. One type of proteasome, the immunoproteasome, which contains inducible catalytic subunits, is adapted to generate peptides for antigen presentation. It has recently been shown that mutations and polymorphisms in the immunoproteasome catalytic subunit *PSMB8* are associated with several inflammatory and autoinflammatory diseases including Nakajo-Nishimura syndrome, CANDLE syndrome, and intestinal *M. tuberculosis* infection. This comprehensive review describes the disease-related polymorphisms in proteasome genes associated with human diseases and the physiological modulation of proteasome function by these polymorphisms. Given the large number of subunits and the central importance of the proteasome in human physiology as well as the fast pace of detection of proteasome polymorphisms associated with human diseases, it is likely that other polymorphisms in proteasome genes associated with diseases will be detected in the near future. While disease-associated polymorphisms are now readily discovered, the challenge will be to use this genetic information for clinical benefit.

## 1. Introduction

Over the last decade, significant improvements have been made in genotyping efficiency, sequencing technology, and statistical methodology, providing researchers with better opportunities to define the role of sequence variation in the development of human diseases [1–3]. Many human diseases are now known to have a genetic component. All humans start their lives with germ-line mutations inherited from their parents. However, the human genetic code is constantly subjected to mutations which can happen during cell division or after exposure to environmental factors such as UV radiation, chemicals, or viruses. These mutations can result in proteins with altered functions, malformed proteins, or even missing proteins. Some of these changes that occur due to a particular mutation have no effect on biological function (silent mutations), some may be beneficial, and some may lead to disease. These genetic variations are important for genetic diversity within the population.

Genome-wide association (GWA) studies have identified alleles related to complex disorders; however some of these alleles seem to be associated with the disease only in certain populations. Most investigations use dense maps of single-nucleotide polymorphisms (SNPs) as well as the haplotypes derived from these polymorphisms. Determining the underlying causal relationship between SNP or haplotype and disease is currently a major challenge. Polymorphisms (termed “alleles”) occur more often (frequency of 1% or greater) in the general population than mutations [4, 5]. Single-nucleotide polymorphisms (SNPs) are the most common type of polymorphism and account for 90% of human DNA polymorphisms. Most SNPs have two alleles which are designated “major” and “minor” based on their observed frequency in the general population. At each SNP, several genotypes are possible because chromosomes are both maternal and paternal in origin: homozygous for the major allele, heterozygous, or homozygous for the minor allele. It is estimated that more than 10 million SNPs occur in our whole genome (once every 100~300 bases) [6]. Because of the large number of SNPs in the whole genome, investigation of all the SNPs for a large number of individuals is time-consuming and costly. Whole genome sequencing for large sample numbers is also not desirable because many SNPs are rare and occur only once (“singletons”) or twice (“doubletons”) in the analyzed samples.

The haplotype refers to an individual collection of short tandem repeat allele mutations at adjacent locations (loci) that are inherited together. Genome scan approaches to find regions associated with diseases are now much more efficient due to efforts such as the HapMap [6]. The HapMap contains maps of haplotype blocks and their SNPs, allowing users to select a group of SNPs to investigate a possible association between known genomic regions and the disease being studied. Smaller research labs are now able to analyze multiple genes belonging to the same pathway instead of analyzing a single polymorphism on a single gene. These advances have led to the discovery of new polymorphisms on proteasome genes that are linked to major human diseases.

### 1.1. The Ubiquitin-Proteasome System (UPS)

**Figure 1:**
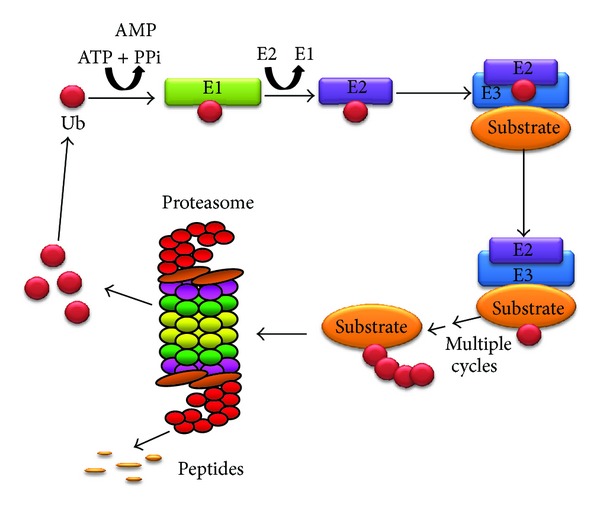
Schematic diagram of the ubiquitin-proteasome system. The UPS involves at least three enzymes (E1, E2, and E3) that catalyze the addition of ubiquitin to lysine residues on the substrate protein. Polyubiquitinated substrates are then recognized by the proteasome or proteasome associating protein, and the ubiquitin removed by deubiquitinases and the substrate unfolded and translocated in the 20S core for proteolysis.

The UPS is the major pathway for degrading intracellular proteins. The number of cellular processes that the UPS system is involved in is impressive and includes cell cycle regulation, cellular differentiation, removal of abnormal and misfolded intracellular proteins, and generation of antigenic peptides [7–10]. The first step in UPS-mediated protein degradation involves ubiquitination, which acts as a signal for degradation and is carried out by a series of enzyme-mediated reactions involving at least three types of enzymes, E1, E2, and E3 (Figure 1). The ubiquitin-activating enzyme (E1) generates activated ubiquitin (Ub) via an ATP-dependent mechanism. Activated Ub is transferred to the ubiquitin-conjugating enzymes (E2), which, together with ubiquitin protein ligases (E3), ligates Ub to lysine residues on protein substrates [11]. This process of ubiquitination occurs multiple times resulting in ubiquitinated substrates which are recognized by the proteasome or proteasome associated proteins. Once bound to the proteasome, the polyubiquitin tag on the substrate is removed by deubiquitinases which allows the Ub to be recycled in the cell. The deubiquitinated substrate is then unfolded and translocated into the 20S core by the 19S regulatory particle. Once inside the core, the substrate is degraded by the proteolytic enzymes of the 20S proteasome. The proteasome contains three proteolytic activities: caspase-like (*β*1i), trypsin-like (*β*2i), and chymotrypsin-like (*β*5i) activity.

The importance of the proteasome in cellular functions is exemplified by experimental evidence which suggests that the proteolytic capacity of the proteasome in certain tissues declines with age and that this decline in proteasome activity is related to the lifespan of the organism [12–16]. In contrast, long-lived naked mole rats and centenarians show elevated proteasome levels and activity [15, 16]. Aging cells have increased levels of damaged proteins, possibly increasing the load on the proteasome [17]. This proposed imbalance between proteasome activity and proteasome substrate load has been suggested to be responsible for the buildup of protein aggregates in aged cells. The impact of proteasome proteolytic capacity on the replicative lifespan in *Saccharomyces cerevisiae* was investigated using a genetic system that allowed the abundance of UPS components to be manipulated at the transcriptional level [18]. Increasing the levels of the UPS-related transcription factor Rpn4 upregulates UPS components and enhances replicative lifespan and resistance to proteotoxic stress. This effect of increased proteasome capacity on lifespan is independent of the proteotoxic stress response [18].

In a yeast model for neurodegenerative diseases, elevated proteasome capacity results in improved clearance of toxic Huntington fragments, suggesting that lifespan extension may be related to elimination of damaged proteins in old cells [18]. Overexpression of the proteasomal deubiquitinating subunit Rpn11 extends lifespan in flies [19]. In *C. elegans* the downregulation of proteasome regulatory particle subunits leads to a substantial shortening of lifespan [20].

### 1.2. Proteasome Components

**Figure 2:**
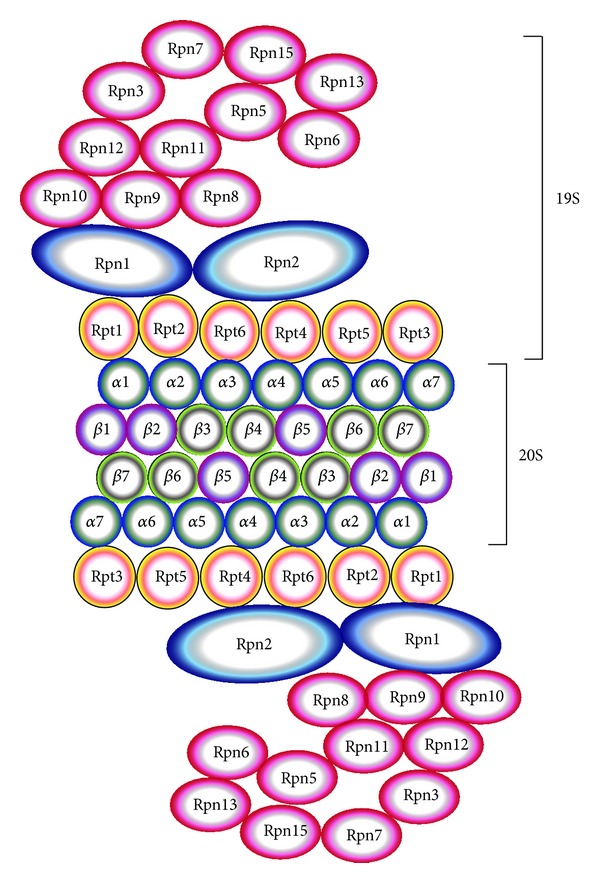
Schematic diagram of the 26S proteasome. The 26S proteasome is composed of the core 20S proteasome and the 19S proteasome complex.

**Figure 3:**
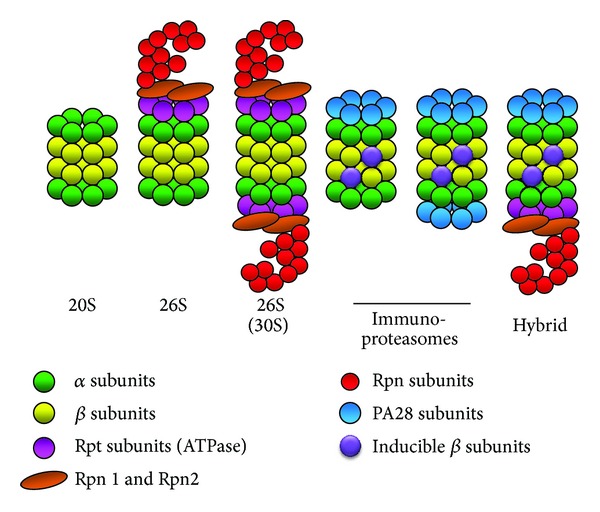
Schematic diagram of different forms of the proteasome. Intracellular proteasome exists in different forms. The 26S proteasome can exist with one or two 19S caps, immunoproteasomes containing one or two 11S caps, proteasomes containing the 20S proteasome with one or two PA200 caps (in the nucleus only), and hybrid proteasomes which contain different combinations of 20S and activators.

The proteasome is a multicatalytic enzyme which is highly conserved. The predominant intracellular form is composed of two large complexes, the 20S and 19S complexes (Figure 2) [21–23]. Proteasomes are found in archaebacteria as well as the nucleus and cytoplasm of all eukaryotic organisms. The proteasome complex is essential for cellular processes, as removal of any proteasome gene is lethal in eukaryotes [24, 25]. The 20S proteasome, or core particle, contains the proteolytic sites responsible for protein degradation. The 20S proteasome is a 28-subunit barrel-like structure of four rings of subunits (two *α* and two *β* rings, arranged *αββα*), with each ring containing seven subunits. Each *α* and *β* subunit occurs in duplicate and three of the *β* subunits have proteolytic capabilities: *β*1 (encoded by *PSMB6* gene), with caspase-like proteolytic activity; *β*2 (*PSMB7*), with trypsin-like activity; and *β*5 (*PSMB5*), with chymotrypsin-like activity. The gene and protein names of the components of the proteasome are shown in Table 1. The 19S proteasome complex, or regulatory complex, is important in mediating substrate recognition, processing, and transporting substrates into the catalytic chamber of the 20S core [26]. Modulation of the 20S and 26S proteasomes by posttranslational modifications has been shown to affect proteasome activity [27]. The 19S is structurally more complex than the 20S, with six different ATPases that unfold globular proteins, non-ATPase regulatory subunits that bind polyubiquitin chains, and non-ATPase regulatory subunits that cleave ubiquitin moieties off of polyubiquitinated proteins. The ATP-dependent 19S regulatory complexes are involved in unfolding and translocating polyubiquitinated substrates into the interior of the 20S complex. Once inside the 20S core substrates are degraded into oligopeptides. While ATP hydrolysis is not needed to cleave the substrate peptide bonds, ATP is needed for substrate unfolding and translocation into the proteasome's 20S core chamber. The 19S proteasome can be replaced by other proteasome activator complexes (also called 11S, encoded by *PSME* genes), or PA200 (*PSME4*) [28–30]. PI31 (*PSMF1*) inhibits the activation of the 20S proteasome by 19S and 11S and inhibits hydrolysis of protein and peptide substrates by the 20S proteasome [31, 32]. Intracellularly, multiple forms of the proteasome with different combinations of activators coexist (Figure 3). These different forms have different proteolytic activities and functions and are likely to be an important contributing factor in diseases.

Two other 20S proteasome genes, *PSMA8* and *PSMB11* (codes for *β*5t), which occur in specific tissues, were recently reported but are not currently known to be associated with any diseases [33, 34]. In mammalian testis, most proteasomes contain a spermatid/sperm-specific *α* subunit *PSMA8* and the PA200 activator [33]. These mammalian testis proteasomes, called spermatoproteasomes, are important for the polyubiquitin-independent degradation of histones. Another catalytic proteasome subunit, *β*5t, was found to be expressed exclusively in cortical thymic epithelial cells [34]. The replacement of *β*5 or *β*5i with *β*5t selectively reduces chymotrypsin-like activity of the proteasome [34]. The thymoproteasome (proteasome with *β*5t) is important for development of CD8(+) T cells in the thymus, as it plays a key role in generating the MHC class I-restricted CD8(+) T cell repertoire during thymic selection [34, 35].

## 2. 20S Proteasome Mutations and Polymorphisms

**Figure 4:**
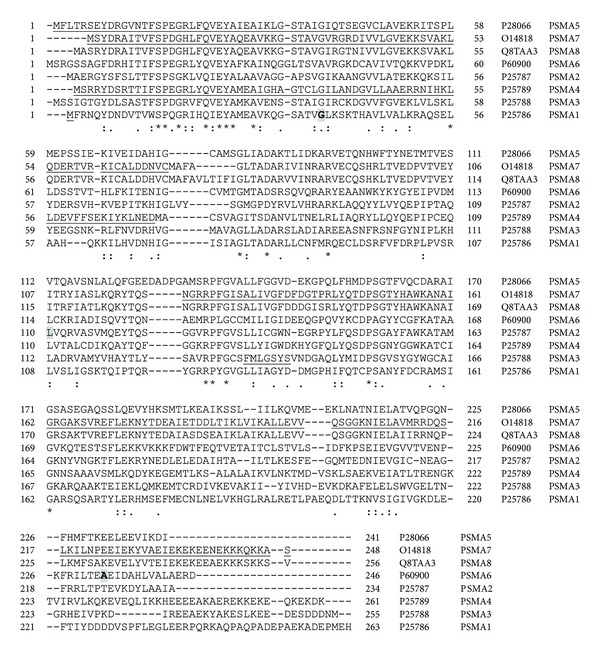
Alignment of human PSMA subunits 1–8. Protein sequences of the eight proteasome PSMA subunits were aligned using Clustal W. ⋆, identical residue in all seven subunits;  :, conserved amino acids with strongly similar properties; ., conservation between residues of weakly similar properties. Naturally occurring variants are highlighted with grey boxes. Alternatively spliced regions are underlined. Amino acid residue numbers are shown on the left and right of each sequence and the UniProt accession number and gene name of each sequence are shown to the right of each sequence. PSMA8 (PSMA7L) is found only in mammalian testis and is a spermatid/sperm-specific *α* subunit [33].

**Figure 5:**
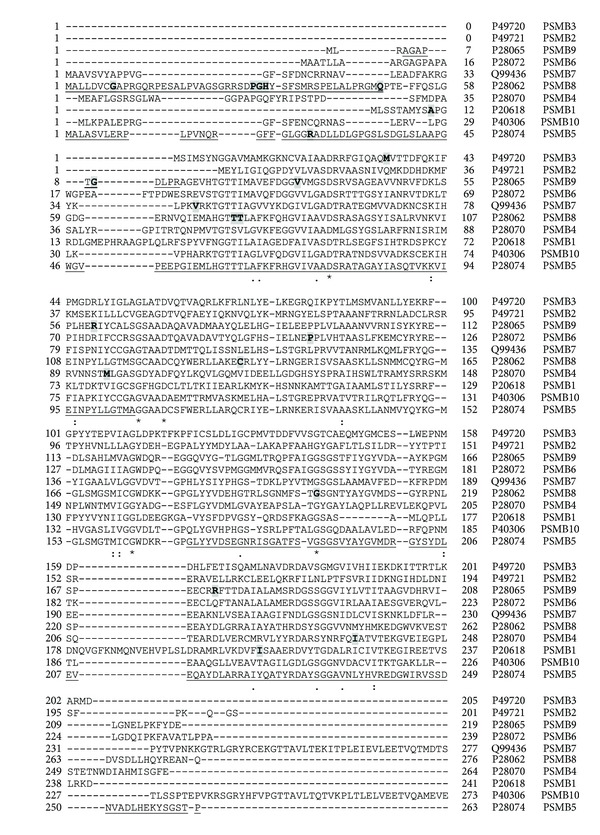
Alignment of human PSMB subunits 1–10. Protein sequences of the ten proteasome PSMA subunits were aligned using Clustal W. ⋆, identical residue in all ten subunits;  :, conserved amino acids with strongly similar properties;  ., conservation between residues of weakly similar properties. Naturally occurring variants are highlighted with grey boxes. Alternatively spliced regions are underlined. Amino acid residue numbers are shown on the left and right of each sequence and the UniProt accession number and gene name of each sequence are shown to the right of each sequence.

**Figure 6:**
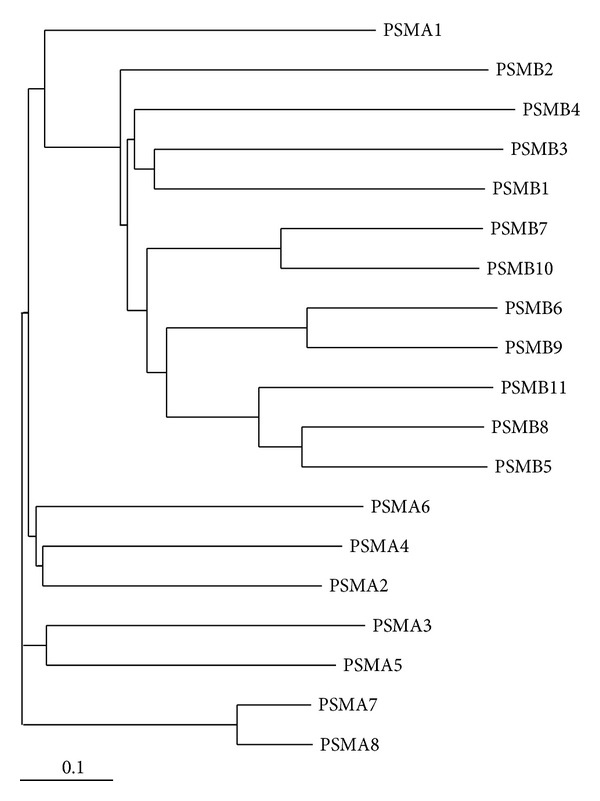
Phylogenetic tree of human 20S proteasome subunits. Phylogenetic tree was generated using Clustal W2 phylogeny [88] and image obtained using TreeView [89]. The UniProt accession numbers used for the alignment of proteasome subunits are given in Figures 4 and 5.

While all *PSMA* and *PSMB* genes have known gene mutations [38], only a few 20S proteasome genes have detected polymorphisms that are associated with disease.  Table 2 shows the polymorphisms in proteasome genes that are associated with diseases. Alignment of human *PSMA* (Figure 4) and human *PSMB* (Figure 5) protein sequences shows that *PSMA* and *PSMB* proteins have some homology with each other. Phylogenic analyses of *PSMA* and *PSMB* protein subunits show that all subunits are evolutionarily related to each other (Figure 6). Current evidence suggests that two constitutive 20S genes, *PSMA6* and *PSMA7*, have polymorphisms associated with human diseases.

### 2.1. *PSMA6*

The proteasome gene, *PSMA6*, codes for a 246 residue protein called *α*1. This protein is structurally important in forming the outer *α* rings of the 20S core proteasome. The *α*1 protein function is also likely to be modulated by posttranslational modifications including phosphorylation, glycosylation, and lysine acetylation [60, 84]. In humans, *PSMA6* is most closely related to *PSMA4* and *PSMA2* (Figure 6). The location of the *PSMA6* gene occurs in a region containing microsatellites that have been implicated in coronary artery disease (CAD) [85], type 2 diabetes mellitus (T2DM) [86], and Grave's disease [87].

#### 2.1.1. Coronary Artery Disease

No association between two SNPs (rs1048990 and rs12878371) in the *PSMA6* gene, as well as two SNPs in the *KIAA0391* gene and one SNP downstream of both genes, with CAD in a Saudi population (1071 patients and 929 controls) was detected [85]. These two genes, *KIAA0391* and *PSMA6*, which have both been reported to predispose individuals to CAD, form an evolutionarily conserved cluster in the chromosomal region 14q13.2. Interestingly, two haplotypes in the chromosomal region (five SNPs in a 100 kb region of chromosome 14) encompassing *KIAA0391* and *PSMA6* genes, 1A-2G-3C-4A-5A and 1A-2G-3G-4A-5A, show increased risk of both CAD and myocardial infarction (MI), while another haplotype, 1T-2G-3C-4G-5A, showed decreased risk of CAD and MI [85]. These latter results suggest that disease risk factor determination may be improved by investigating haplotypes instead of SNPs. Other recent experimental data suggest that haplotypes are more predictive than individual SNPs at determining risk factors for complex diseases [90].

CAD is a complex disease, and several molecular pathways as well as loci and candidate genes that affect the susceptibility to CAD have been suggested to be involved. A functional sequence variation, −8C/G, in *PSMA6* was found to increase susceptibility to CAD [72]. 713 Caucasian ischemic stroke patients (708 controls) and 166 African American ischemic stroke patients (117 controls) were investigated using odds ratios (ORs) from multivariable logistic regression models for twenty SNPs previously shown to be associated with MI or CAD [72]. The *PSMA6*  −8C > G (SNP rs1048990) was found to have a protective association with ischemic stroke in both Caucasians and African Americans (i.e., decreased risk of ischemic stroke). Investigation of 1330 cases and 2554 controls from Japanese and Korean populations for *PSMA6* genotypes showed no evidence of the association with either population [43]. An investigation of 6946 MI patients and 2720 unrelated controls showed that the homozygous GG genotype for the −8C > G polymorphism was less frequent in the UK population (2.1%) than in the Japanese population (8.9%) [91]. No association between the *PSMA6* polymorphism and MI was found in the British population. Another genetic association study on *PSMA6* −8C/G using 210 North Indian CAD patients and 232 controls did not shown any association between the *PSMA6* variant and CAD [92].

#### 2.1.2. Myocardial Infarction

In a case-control association study of 1884 MI Chinese patients and 2643 unrelated controls, genotyping of the *PSMA6*  −8C > G polymorphism showed that this SNP was associated with MI [73]. No relationship between *PSMA6* −8C > G and sex, age, or other conventional cardiovascular risk factors was detected. A recent meta-analysis of 15,991 cases and 16,784 controls from ten case-control studies suggest that the −8C/G sequence variation is a risk factor for increased CAD susceptibility, but the association between the sequence variation and CAD varies in different ethnic populations [60]. Subgroup analysis of the −8C/G polymorphism data showed increased risks of CAD in East Asians, with no significant associations among other ethnic populations. Subgroup analysis also showed increased risks of MI in all populations.

#### 2.1.3. Type 2 Diabetes Mellitus

Interestingly, the same −8C > G variant of *PSMA6* gene that was associated with CAD was found to be associated with T2DM and diabetes-related metabolic traits in two Chinese populations [74]. 73 Caucasian patients with MI and 151 controls genotyped for variants of the *PSMA6* gene revealed no association between *PSMA6*  −8C > G and MI [75]. However, 34 diabetic subjects with MI showed a significant association with *PSMA6*  −8C > G gene frequency compared to 85 controls [75]. Biopsy specimens taken from the ischemic left ventricle of several patients showed Ub levels and proteasome 20S activity which significantly correlated with plasma glucose levels, with T2DM patients having higher Ub levels and proteasome 20S activity than nondiabetics [75]. This experimental data suggest that the *PSMA6*  −8C > G polymorphism contributes to MI susceptibility in T2DM, possibly by upregulation of the UPS. The *PSMA6*  −8C > G polymorphism was also reported to be associated with lower survival rates in multiple myeloma patients [93].

#### 2.1.4. Graves' Disease

Graves' disease is an autoimmune thyroid disease characterized by hyperthyroidism due to circulating autoantibodies. It is one of the most common thyroid problems and several immune and thyroid related genes appear to influence susceptibility to Graves' disease [94]. A 270 kb chromosome region (14q13.2-14q13) containing *PSMA6* was analyzed for polymorphisms and associations of five microsatellite repeats in 50 Latvian patients with Graves' disease and 116 controls with Graves' disease [87]. Some particular alleles of HSMS006 and HSMS801 (microsatellite polymorphisms) were found more often while some alleles of HSMS006 were found less frequently in Graves' patients when compared to healthy controls. The HSMS602 allele was found in Graves' patients but not in healthy controls [87]. Further analysis is needed to confirm the importance of *PSMA6* in Graves' disease.

#### 2.1.5. Psoriasis

Several psoriasis susceptibility loci have now been detected [95]. A meta-analysis of two GWA studies involving 1,831 cases and 2,546 controls gave 102 potential loci. A three-stage replication study using 4,064 cases and 4,685 controls from Michigan, Toronto, Newfoundland, and Germany found three genomic regions, including one that contains *PSMA6 *and *NFKBIA* (rs12586317) that showed psoriasis susceptibility. The SNP rs12586317 was strongly associated with the subphenotypes of psoriatic arthritis and purely cutaneous psoriasis [95].

### 2.2. PSMA7

The proteasome gene *PSMA7* codes for *α*4, a 248 residue protein that is similar to *PSMA6* in structure. It is also posttranslationally modified by phosphorylation, glycosylation, and lysine acetylation [60, 84, 96]. Phosphorylation of *α*4 at Tyr 153 impaired G1/S transition and S/G2 progression in cells, suggesting that tyrosine phosphorylation of the *α*4 proteasome subunit is important in intracellular regulatory control [96].

#### 2.2.1. Intellectual Disability

Sequencing the coding regions of more than 21,000 genes from 100 patients with an IQ below 50 and their unaffected parents identified 79 *de novo* mutations (affecting 77 genes) in 53 of 100 patients [45]. A *de novo* heterozygous 335C-A transversion in *PSMA7*, resulting in an A112D mutation, was identified in a male patient with severe intellectual disability [45], suggesting that *PSMA7* may be a candidate intellectual-disability gene.

## 3. Immunoproteasome Mutations and Polymorphisms

Specialized proteasomes called immunoproteasomes (Figure 3) are capable of cleaving substrates to generate short peptide fragments that are recognized as antigens in lymphocytes [23, 29, 97]. These antigens are presented on the surface of these cells (through the MHC complex) and play an important role in the cell's ability to mount a specific immune response [97]. When an infection occurs, the hormone interferon is excreted locally, resulting in gamma-interferon inducible beta subunits which replace the constitutive beta subunits. In many eukaryotic immunoproteasomes the 19S complex is replaced by another complex, the PA28 (or 11S) complex. The cytosolic PA28 complex is composed of a six-member ring of PA28*α* and PA28*β* subunits which are products of *PSME1* and *PSME2* genes, respectively. Nuclear immunoproteasomes contain a PA28*γ* complex (*PSME3* gene). The PA28 complexes (caps) are significantly smaller than the 19S complexes but are more efficient at generating antigen peptides. They degrade proteins in an ATP-independent manner unlike the 26S proteasome [97, 98]. Two immunoproteasome genes, *PSMB8* and *PSMB9*, have been shown to be associated with human diseases. Surprisingly, no polymorphisms in the genes connected to the constitutive proteolytic activities of the proteasome (*PSMB5*, *PSMB6,* and *PSMB7*) have been found to be associated with disease. It is possible that polymorphisms in *PSMB5*, *PSMB6,* or *PSMB7* that result in decreased proteasome activity may be severe enough that they cause embryonic lethality.

### 3.1. *PSMB8*

*PSMB8* (proteasome subunit *β* type 8) gene expression is induced by interferon-*γ* (IFN-*γ*), resulting in the upregulation of the protein product of this gene, *β*5i, which replaces the constitutive catalytic subunit *β*5 (*PSMB5*) [99]. The human *β*5i is expressed as a 276 residue protein which requires the proteolytic removal of 72 residues to generate a mature subunit [100]. Although the *β*5i propeptide is not essential for incorporation into the 20S proteasome, presence of this sequence increases the efficiency of *β*5i incorporation and proteasome maturation [101]. Two alternative spliced forms of human *β*5i have been detected, but both forms would result in the same mature protein, as the alternative splicing occurs in the propeptide which is missing in the mature form of *β*5i. The replacement of *β*5 by *β*5i increases the ability of the immunoproteasome to cleave peptides after hydrophobic and basic residues. Mice lacking the *PSMB8* gene had reduced levels of MHC class I cell-surface expression and inefficiently presented the endogenous antigen HY [102]. A selective inhibitor of *β*5i, ONX-0914 (previously referred to as PR-957), blocked presentation of MHC class I-restricted antigens *in vitro* in splenocytes and *in vivo* in mice [103]. In mouse models, inhibition of *β*5i reversed signs of rheumatoid arthritis and reduced cellular infiltration, cytokine production, and autoantibody levels, suggesting that *β*5i has an important role in regulating pathogenic immune responses [103].* PSMB8* has recently been shown to have a role in cytokine production [103]. ONX-0914 blocked the production of IL-6, IL-23, and TNF-*α* by *∼*50% or greater [103]. ONX-0914 also ameliorated disease in two mouse models of arthritis [103].

**Figure 7:**
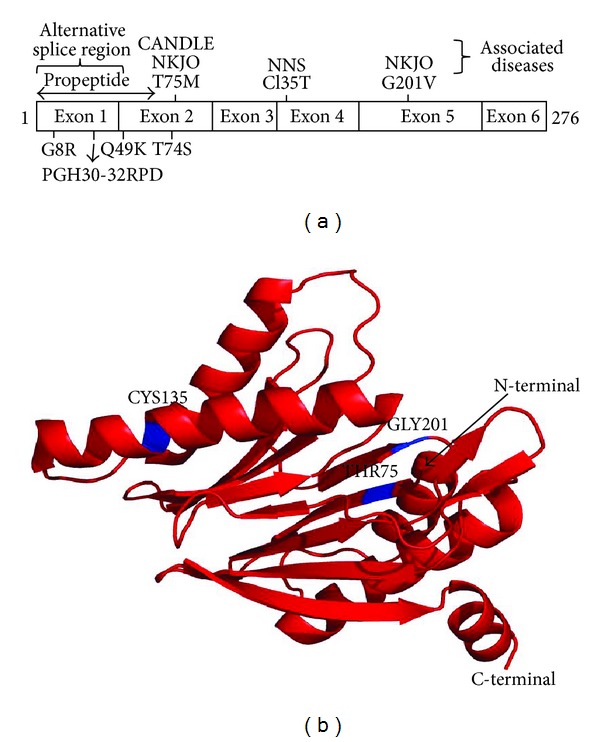
Schematic diagram of PSMB8 showing the location of known polymorphisms. (a) Diagram of PSMB8 showing exon organization (drawn to relative scale), location of alternative spliced region, propeptide region that is removed in the mature form of the protein, location of disease causing polymorphisms', and location of other known polymorphisms. (b) Tertiary structure of *β*5i *(PSMB8)* showing polymorphisms (shown in blue) associated with diseases. Structure created using PyMol (http://pymol.org/).

**Figure 8:**
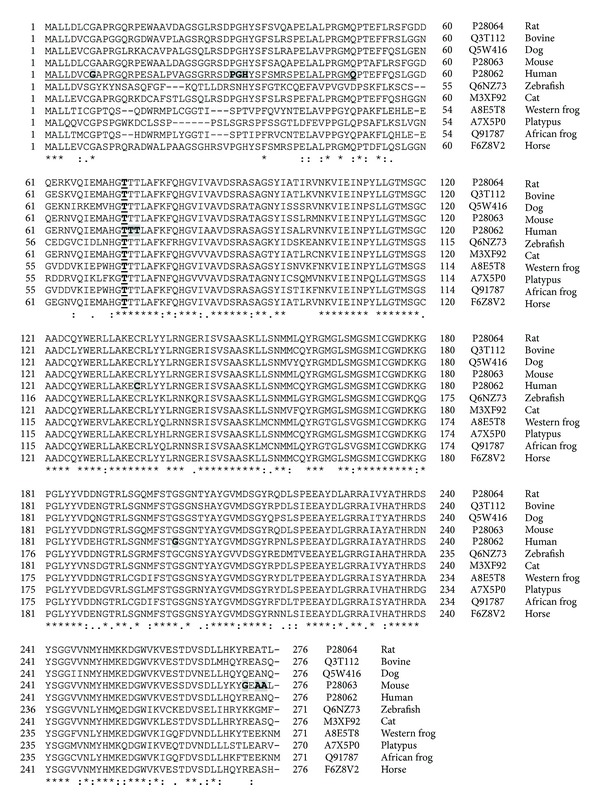
Sequence alignment of human PSMB8 from different animals. Protein sequences of *Rattus norvegicus* (rat), *Bos taurus* (bovine), *Canis familiaris* (dog), *Mus musculus* (mouse), *Homo sapiens* (human), *Danio rerio* (Zebrafish), *Felis catus* (Cat), *Xenopus tropicalis* (western clawed frog), *Ornithorhynchus anatinus* (Duckbill platypus), *Xenopus laevis* (African clawed frog), and *Equus caballus* (Horse) PSMB8 were aligned using Clustal W. ⋆, identical residue in all six subunits;  :, conserved amino acids with strongly similar properties;  ., conservation between residues of weakly similar properties. Naturally occurring variants are highlighted with grey boxes. Alternatively spliced regions are underlined. Amino acid residue numbers are shown on the left and right of each sequence and the UniProt accession number is also shown.

Genetic variants of *PSMB8* are associated with the development of many diseases, including viral infection, autoimmune disease, and malignant tumors.  Figure 7 shows a schematic diagram of the well-annotated polymorphisms as well as the exon organization of *PSMB8*. The structure of the protein product of *PSMB8* showing the location of the three residues associated with diseases is also shown in Figure 7. The residues that are mutated by the disease-associated polymorphisms in *PSMB8* are highly conserved from zebrafish to man (Figure 8).

#### 3.1.1. JMP Syndrome

JMP syndrome (autosomal-recessive autoinflammatory syndrome characterized by joint contractures, muscle atrophy, microcytic anemia, and panniculitis-induced lipodystrophy) patients show hepatosplenomegaly and hypergammaglobulinemia as well as lipodystrophy of the arms, face, and thorax. Using genome-wide homozygosity mapping, a homozygous missense mutation (c.224C > T, Thr75Met) in the proteasome gene *PSMB8* that encodes the *β*5i (LMP7) subunit was detected in two pedigrees from Portugal and Mexico with JMP syndrome [54]. Segregation of this mutation in other members of the pedigrees occurred in an autosomal-recessive fashion. Measurement of proteasome activity in the cell lysates of Epstein-Barr virus-transformed lymphoblasts from a control subject and an affected (T75 M) patient showed reduced chymotrypsin-like activity, but similar trypsin-like and caspase-like activity in the affected patient relative to the control subject. Serum from two affected patients showed 2.8- to 3.5-fold, 1.6- to >9-fold, and 7- to 19-fold increased levels of interferon *γ*, IL-8, and IL-6 respectively. These results and other results from these patients, such as increased serum *γ* globulins and erythrocyte sedimentation rate without elevation in other cytokines such as IL-1 and TNF-*α*, suggest significant ongoing inflammation and a potentially unique biomarker signature in JMP syndrome patients.

#### 3.1.2. Nakajo-Nishimura Syndrome

Nakajo-Nishimura syndrome (NNS) was first reported by Nakajo in 1939 [104]. NNS is a rare, distinct inflammatory, and wasting disease which usually begins in early infancy and has only been reported in Japanese patients [105]. Clinical features of this disease include elongated and thickened fingers, hereditary lipomuscular atrophy with joint contractures, periodic high fever, hyper-*γ*-globulinemia nodular erythema, and myositis [106, 107]. Extracts separated by glycerol gradient centrifugation from immortalized lymphoblastoid cell lines obtained from an NNS patient, his heterozygous parent, and a healthy control showed that all three proteolytic activities of the proteasome were decreased in the NNS patient relative to the healthy control. Due to the low number of samples investigated, the results should be viewed with caution but do suggest that the G210V mutation is associated with decreased immunoproteasome activity. NNS cells also show an accumulation of immature 20S proteasome precursors before incorporation of *β*5i into the complex. In silico modeling of the G210V suggests that this assembly defect could be due to the proximity of *β*5i, *β*4, and *β*6 next to each other resulting in conformation changes in both Thr73 and Lys105 which are part of the catalytic center of *PSMB8*. Interestingly, some of the G210V mutant *β*5i subunits incorporated into the mature proteasome appeared to be insufficiently cleaved. These results suggest that the G210V mutation affects both *β*5i catalytic activity and assembly of the 20S proteasome. A polymorphism in *PSMB8* resulting in a Q49 K amino acid change in *β*5i was found to be associated with juvenile rheumatoid arthritis [108]. Some of the features of juvenile rheumatoid arthritis are similar to NNS.

#### 3.1.3. Candle Syndrome

Chronic atypical neutrophilic dermatosis with lipodystrophy and elevated temperature (CANDLE syndrome) is a recently described autoinflammatory syndrome [77]. Autoinflammatory diseases differ from autoimmune diseases in that they are primarily a result of alterations in the innate immune system instead of perturbations in adaptive immunity [109]. Patients with CANDLE syndrome typically show recurrent fevers, hypochromic or normocytic anemia, delayed physical development, and variable clinical features including acanthosis nigricans (skin hyperpigmentation), alopecia areata (spot baldness), and hypertrichosis (werewolf syndrome) [109, 110]. A recent genome-wide analysis of nine affected patients in eight families suggests that mutations in *PSMB8* may be the molecular basis of CANDLE syndrome [55]. Four patients were homozygous and two were heterozygous for a missense mutation (c.224C > T), two patients were homozygous for a nonsense mutation in *PSMB8* (c.405C > A), and one patient showed no mutation. None of these sequence changes were observed in chromosomes from 750 healthy controls. Only two of the four patients with the same mutation shared the same haplotype, indicating a mutational hot spot.

#### 3.1.4. Bacterial Infection

*Mycobacterium tuberculosis* (*M. tuberculosis*) infection is a common bacterial infection that is the leading cause of morbidity and mortality compared to all other infectious agents [111]. Extrapulmonary tuberculosis, which is common in the intestinal tract, bones, kidney, lymph gland, skin, and other organs, occurs in 5–20% of all tuberculosis cases and is increasing in both developed and developing countries [112]. Using PCR-based restriction digest, sequencing of digests, and logistic regression analysis, a study involving 168 Chinese patients with intestinal tuberculosis and 235 normal controls identified a polymorphism in *PSMB8* (Q145 K) which were found to be associated with intestinal *M. tuberculosis* infection [78]. *M. tuberculosis* antigenic peptides are produced by the immunoproteasome and subsequently presented on the cell surface by the MHC-I molecule resulting in CD8+ cytotoxic T lymphocytes eliminating *M. tuberculosis* infected cells. Mice lacking the three immunoproteasome catalytic subunits showed defects in presenting several major histocompatibility complex (MHC) class I epitopes in dendritic cells [113]. During viral infection *in vivo*, the MHC class I-presented peptides in immunoproteasome-deficient animals were significantly reduced compared with wild-type mice, whereas presentation of MHC class II peptides was unaffected. These reductions in MHC class I-presented peptides and changes in the type of class I-presented peptides caused transplant rejection of wild-type cells in mutant mice [113].

#### 3.1.5. Cancer

A high risk of colon cancer was associated with a LMP7-K/Q genotype (*PSMB8*) while a low risk was associated with the LMP7-Q/Q genotype in an investigation of 112 colorectal carcinoma patients and 62 control patients [79]. Stimulation of colon carcinoma cell lines with interferon (IFN)-*γ* exhibited a 10-fold increase in LMP7-Q transcript amounts, but only 3.8-fold increase in LMP7-K [79]. The LMP7-K allele showed reduced transcript stability compared with LMP7-Q. Overall, the LMP7-K allele seems to reduce the immunoproteasome formation which results in reduced peptide processing and reduced peptide-HLA presentation [79]. Peptide-HLA presentation is a crucial factor in the immune response against cancer. Immunoproteasomes generate immunogenic tumor peptides which are important for the destruction of cancer cells by cytotoxic T lymphocytes.

#### 3.1.6. Ankylosing Spondylitis

Ankylosing spondylitis (AS) is an inflammatory rheumatic disease which affects men more often than women and is strongly associated with human leucocyte antigen (HLA)-B27 and with the fusion of the spine vertebrae [114]. Inflammation of the joints is common in AS but other parts of the body, such as eyes and bowels, can also show inflammation. The first study to suggest that the *PSMB8* gene was associated with AS involved 57 AS patients and 102 matched random controls [80]. This investigation found that the HLA-B27 polymorphism in *PSMB9* and LMP7-Q/Q (*PSMB8*) confers a higher relative risk than with HLA-B27 alone. A significant association was observed between the LMP7-Q/Q genotype and AS.

#### 3.1.7. Type 1 Diabetes Mellitus

A genomic polymorphism (G/T-37360) in *PSMB8* was strongly associated with type 1 diabetes mellitus (T1DM) in an investigation of 198 unrelated T1DM Caucasian patients and 192 normal Caucasian controls from the southeastern United States [81]. The R/H-60 polymorphism in *PSMB8* was found to be associated with T1DM only in subjects containing an HLA DR4-DQB1*0302 haplotype. However, results from this same study suggest that *PSMB8* genes have independent effects on T1DM susceptibility [81].

Some of the clinical features of *PSMB8* mutations may be due to the role of *PSMB8* in autophagy as *PSMB8* seems to play a key role in apoptosis. IFN-*γ* causes increased sensitivity to apoptosis in atherosclerotic lesions. IFN-*γ* sensitized cells from the fibrous cap of human atherosclerotic lesions showed reduced Mcl-1, phospho-Bcl-2 (S70), and phospho-Bcl-X(L) (S62) protein levels. Knockdown of *PSMB8* with siRNA protected the antiapoptotic protein Mcl-1 from degradation [115]. These results suggest that the immunoproteasome may be a key link between inflammatory factors and the control of vascular cell apoptosis [115].

### 3.2. *PSMB9*

Like *PSMB8* expression, *PSMB9* gene expression is induced by IFN-*γ*, resulting in the upregulation of the protein product of this gene *β*1i, which replaces the constitutive catalytic subunit *β*1 (*PSMB6*) [99]. The human *β*1i is expressed as a 219 residue protein which requires the proteolytic removal of 20 residues to generate a mature subunit. Although two alternative transcripts which encode different isoforms have been reported (Figure 5), the alternative splicing occurs in the region of *β*1i (propeptide) that is removed in the mature form, resulting in the same mature protein. Upregulation of MHC-linked *β*1i and *β*5i subunits amplifies specific endopeptidase activities of the proteasome resulting in the increased production of peptides which terminate almost exclusively with hydrophobic or basic residues, such as those found on MHC class I molecules [99, 116].

*β*1i-deficient mice, generated by replacing a 800 bp region of the *PSMB9 *gene with a neomycin resistance gene in embryonic stem cells, were viable, and healthy with no gross anatomical abnormalities [117]. Purified proteasomes from the spleen and liver of *β*1i-deficient mice showed lower peptidase activity against hydrophobic and basic substrates (but not acidic substrates) when compared to purified proteasomes from wild-type tissues. Antigen-presenting cells from *β*1i-deficient mice displayed reduced ability to stimulate a T-cell hybridoma specific for a nucleoprotein envelope antigen of an influenza A virus [117]. *β*1i-deficient mice also showed only 60%–70% of wild-type levels of CD8-positive T lymphocytes and generated fewer influenza nucleoprotein-specific cytotoxic T lymphocyte precursors. Hence *β*1i is important in antigen processing of MHC class I-restricted antigens.

#### 3.2.1. Graves' Disease

Several investigations demonstrated potential associations between codon 60 R/H polymorphism in *PSMB9* (p.60R > H; c.179G > A; rs17587) and increased susceptibility to various diseases. This *PSMB9* genetic R/H polymorphism at codon 60 had H allele frequencies of 1.1% to 34%, depending on ethnic group [118, 119]. DNA from 306 Caucasian patients with Graves' disease and 364 Caucasian control subjects were investigated for the distribution of an R/H polymorphism in the *PSMB9* gene and a G/T polymorphism in the *PSMB8* gene [82]. The R allele and the RH genotype were increased in subjects with Graves' disease when compared with control subjects. Independently, DNA from 129 families, including parents, an affected sibling with Graves' disease, and an unaffected sibling, were investigated. No preferential allelic transmission occurred from heterozygote parents to offspring at either locus, suggesting that the association of the R/H polymorphism at codon 60 of *PSMB9* with Graves' disease is due to linkage disequilibrium with the associated HLA haplotype [82].

Mishto et al., 2006, [120] found that the codon 60 R/H polymorphism results in a decreased chymotrypsin-like proteasome activity in the aged brain, while recombinant peptides mimicking endogenous substrates showed no differences in the substrate hydrolysis profiles between the codon 60 genotypes [121]. Using fluorogenic substrates that are hydrolyzed selectively by *β*1i, measurement of *β*1i catalytic activity showed that the codon 60 R/H polymorphism did not alter the activity of *β*1i among the cancer cell lines tested [122]. Western blotting showed that the levels of *β*1i were highly elevated in clinical colon cancer tissues compared to the paired nonmalignant colonic tissues. These effects all suggest inconsistent results regarding the influence of this polymorphism on proteasome activity.

#### 3.2.2. Colorectal Cancer

Genotyping of 1467 SNPs (in 871 candidate cancer genes) in 2575 Caucasian colorectal cancer patients and 2707 controls indicated an association with 44 SNPs and colorectal cancer [123]. One of these SNPs, rs241419 (V32I) in *PSMB9*, showed a significant association with an increased risk of colorectal cancer. However, validation of rs241419 association with colorectal cancer was not carried out using kin-cohort analysis of first-degree relatives as was carried out for some other SNPs validated [123].

#### 3.2.3. Ankylosing Spondylitis

193 unrelated Caucasians and 49 Chinese B27 individuals with AS were investigated to determine *PSMB9* gene influence on disease susceptibility in HLA-B27 individuals with AS [83]. HLA-B27 typing showed the involvement of the *PSMB9* gene in the expression of AS in B27 individuals. The LMP2BB genotype (*PSMB9*) was investigated in 546 AS patients (41 Caucasians and 17 Mexican) and 4352 controls. The LMP2BB genotype was significantly decreased in Caucasian and Mexican AS patients compared to random Mexican and Caucasian controls, respectively [124].

#### 3.2.4. 19S Proteasome Mutations and Polymorphisms

**Figure 9:**
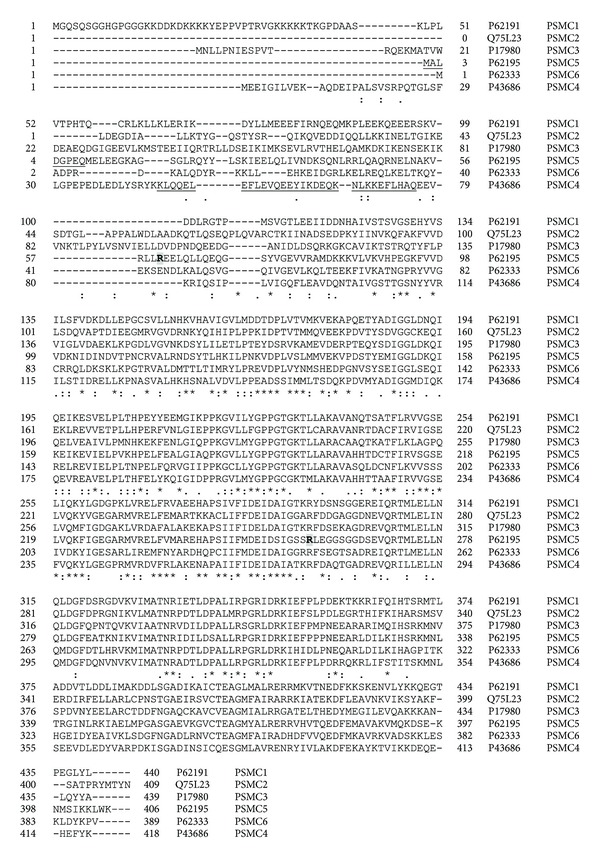
Alignment of human PSMC subunits 1–6. Protein sequences of the six proteasome PSMC subunits were aligned using Clustal W. ⋆, identical residue in all six subunits; :, conserved amino acids with strongly similar properties;  ., conservation between residues of weakly similar properties. Naturally occurring variants are highlighted with grey boxes. Alternatively spliced regions are underlined. Amino acid residue numbers are shown on the left and right of each sequence and the UniProt accession number and gene name of each sequence are shown to the right of each sequence.

**Figure 10:**
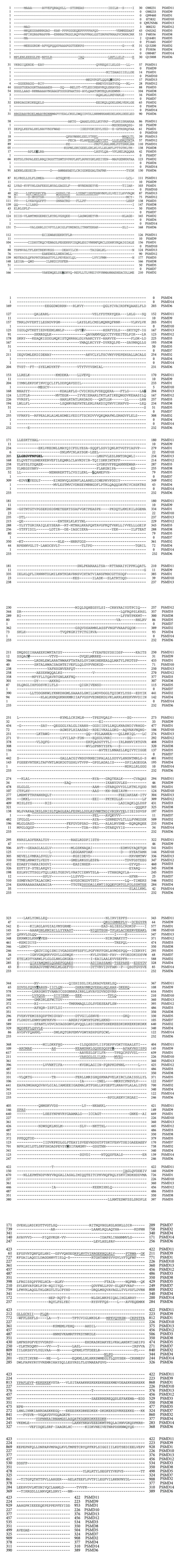
Alignment of human PSMD subunits 1–14. Protein sequences of the thirteen proteasome PSMD subunits were aligned using Clustal W. No residues are conserved in all PSMD subunits. Naturally occurring variants are highlighted with grey boxes. Alternatively spliced regions are underlined. Amino acid residue numbers are shown on the left and right of each sequence and the UniProt accession number and gene name of each sequence are shown to the right of each sequence.

**Figure 11:**
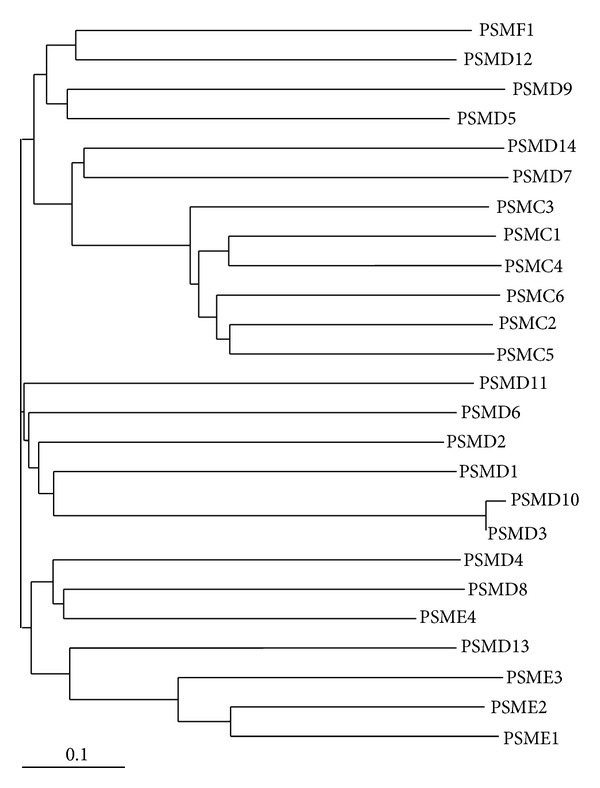
Phylogenetic tree of human PSMC, PSMD, PSME, and PSMF proteasome subunits. Phylogenetic tree was generated using Clustal W2 phylogeny [88] and image obtained using TreeView [89]. The UniProt accession numbers used for the alignment of proteasome subunits are given in Figures 9, 10, 12, and 13.

Alignment of human *PSMC* (Figure 9) and human *PSMD* (Figure 10) shows the relatedness of the 19S proteasome subunits. *PSMF1* shares some homology with *PSMD12* (approximately 40%) while *PSMD8* shares homology with *PSME4* (approximately 40%) (Figure 11). Limited data suggests that four 19S genes, *PSMD3*, *PSMD7*, *PSMD13*, and *PSMD14*, may be associated with human diseases.

### 3.3. *PSMD3*

The *PSMD3* gene encodes a member of the proteasome 19S regulatory cap, Rpn3. Rpn3 is one of the non-ATPase subunits and is composed of 534 amino acids. *PSMD3* variants are associated with insulin resistance in different populations and these relationships are likely to be modified by dietary factors [76]. Insulin resistance is critical in the pathogenesis of chronic diseases such as CAD, hypertension, inflammation, and T2DM [125, 126].

#### 3.3.1. Diabetes as Related to Insulin and Dietary Intake

The UPS has been shown to regulate insulin signal transduction via several mechanisms, including regulation of glucose transporters, ubiquitination of the insulin receptor, and degradation of insulin receptor substrate [127]. Ten SNPs covering 90% the genetic variations in or near *PSMD3* were investigated. Using two independent groups: the GOLDN (Genetics of Lipid Lowering Drugs and Diet Network) study which included 820 participants of Northern European origin, and the BPRHS (Boston Puerto Rican Health Study) study, which included 844 participants recruited by the Boston Puerto Rican Center for Population Health and Health Disparities, the minor C allele carriers of the SNP rs4065321 had a higher homeostasis model assessment of insulin resistance than noncarriers in males of both studies. An interaction between SNP rs709592 and dietary carbohydrate on a higher homeostasis model assessment of insulin resistance subjects with the T allele was detected in the GOLDN group. SNPs rs4065321 and rs709592 both significantly interacted with dietary factors in the GOLDN study.

#### 3.3.2. White Blood Cell Count

Total white blood cell (WBC) and neutrophil counts vary among different ancestry groups and are lower among individuals of African descent [128]. Measuring WBCs in humans is universally used in diseased and asymptomatic patients to identify or predict chronic disease. WBCs are made up mostly of neutrophils, which are a key component of the innate immune system as an early line of defense against invading microorganisms. Very low numbers of neutrophils have been shown to make patients susceptible to bacterial infections and can lead to lethal conditions [129]. *PSMD3* has also been reported to be associated with white blood cell counts [130–132]. The rs4065321 of *PSMD3*-*CSF3* region was associated with WBC count in African American and other populations. GWA analysis of 13,923 subjects in the electronic Medical Records and Genomics (eMERGE) Network identified two regions each unique to subjects of genetically determined ancestry to the African continent or to the European continent [131]. One of these regions contained the *PSMD3* intronic SNP rs4065321 (in persons of European ancestry) that was found to be significantly associated with WBC count [131]. A GWA study in 5771 Japanese and a replication study using independent 1894 Japanese identified rs4794822 in *PSMD3-CSF3* as being significantly associated with neutrophil count [132]. The SNP rs4794822 in *PSMD3-CSF3* was not associated with lymphocyte, monocyte, eosinophil, or basophil counts, suggesting a specific association with neutrophils [132].

### 3.4. *PSMD7*

*PSMD7*, the 19S proteasome non-ATPase regulatory subunit 7, encodes the protein Rpn8, which is involved in the ATP-dependent degradation of ubiquitinated proteins [133]. Rpn8 is a 324 residue protein which is modified by acetylation of K204 and K214 and may be involved in viral replication [84, 134]. The HIV-1 accessory gene product Vpr interacts with MOV34 (homologous to *PSMD7*) [134]. The induction of cell cycle arrest at the G2/M phase border by Vpr correlated with a change in the subcellular localization of MOV34 from a nuclear to a perinuclear localization as well as the inhibition of the maturation promoting factor-associated histone H1 kinase activity. These results suggest that *PSMD7* may be involved in the regulation of the cell cycle and is a likely cofactor for HIV-1 Vpr [134].

#### 3.4.1. Ankylosing Spondylitis

Blood samples from 185 Chinese patients with AS (149 male) and 516 healthy controls (412 male) showed that SNP rs17336700 of *PSMD7* is significantly associated with AS in a Chinese population [18]. Two mutations, 392-187C → T and 392-192delTC, were detected once in the AS patients. The SNP rs17336700 had a minor allele frequency of 13.0% and was significantly increased in AS patients relative to controls. Allele-wise analysis also indicated a higher frequency of the rs17336700 C allele in patients when compared to controls [18]. Human liver biopsy samples from 73 patients (containing eight rs17336700 TC heterozygotes) showed that *PSMD7* gene expression in the TC group was 1.88-fold higher than that of the TT group.

### 3.5. *PSMD13*

*PSMD13* is one of the least understood proteasome genes. It codes for a 376 amino acid protein called Rpn9 which is part of the 19S regulatory cap that is involved in the ATP-dependent degradation of ubiquitinated proteins [135]. Two isoforms of *PSMD13* are produced in humans by alternative splicing and its translated product Rpn11 is acetylated at K298 [84].

#### 3.5.1. Platelet Traits

Platelet traits have been shown to be highly heritable and well established as being important for the pathogenesis of atherothrombosis and cancer. Investigation of genetic variants associated with platelet traits identified five chromosomal regions associated with variation in the number of circulating platelets (PLT) and eight associated with mean platelet volume (MPV) variation with genome-wide significance [136]. Several SNPs near the telomere region of chromosome 11p were associated with PLT. This region contains six genes, including *PSMD13*. Like most complex diseases multiple genetic loci influence interindividual variation in platelet traits.

### 3.6. *PSMD14*

*PSMD14* codes for Rpn11 which is a metalloprotease (binds zinc) that specifically cleaves K63-linked but not K48-linked polyubiquitin chains [137]. As part of the 19S, Rpn11 is involved in the ATP-dependent degradation of ubiquitinated proteins. Rpn11 is a 310 residue protein which is important for recycling Ub from proteasome substrates and is also a key deubiquitinating enzyme for regulating Ub conjugates generated in response to DNA damage as well as several aspects of the mammalian DNA double-strand break response [138]. In *Schizosaccharomyces pombe,* the yeast equivalent of *PSMD14* (POH1), has been shown to confer pleiotropic drug resistance to taxol, doxorubicin, 7-hydroxystaurosporine, and ultraviolet light when transiently overexpressed in mammalian cells [139]. These experimental data all suggest that Rpn11 is important in cellular susceptibility to cytotoxic agents. Rpn11 is known to be phosphorylated on S150 and S224 [140].

#### 3.6.1. Intellectual Disability

*PSMD14* is part of a 0.4 Mb region of 2q24.2 that is associated with intellectual disability and short stature [141]. An 18-year-old male with mild intellectual disability and short stature had a 0.422 Mb deletion on 2q24.2 which was detected by comparative genomic hybridization. This deleted region included three genes: TBR1, TANK, and* PSMD14* [141]. While it is not known if all three genes are important for the phenotype, the association of other proteasome genes with intellectual disability suggests that *PSMD14* is a possible candidate gene that may be associated with intellectual disability. The proteasome is likely involved in intellectual disabilities indirectly by altering the degradation of key signaling proteins important for intellect.

## 4. Polymorphism Associated with Reduced Risk of Disease

### 4.1. Multiple Sclerosis

Multiple sclerosis (MS) is a common but complex autoimmune disease which displays accumulated immunoproteasomes in plaques of affected brain areas. The immunoproteasome *PSMB9* codon 60HH variant was observed to have a reduced risk of developing MS in HLA-A*02+ Italian females [142]. Although the role of the proteasome in autoimmune diseases is only partly understood, the treatment of autoimmune diseases with proteasome inhibitors has been successful in animal models [143, 144]. Production of MHC class I-restricted epitopes by the proteasome is a key step in the activation and regulation of autoreactive CD8+ T cells. Immunoproteasomes carrying the *PSMB9* 60H allele show a lower amount of the HLA-A*0201 restricted epitope myelin basic protein residues 111–119 (MBP_111–119_) *in vitro* [142]. It is possible that the altered proteasome-dependent production of a specific MBP epitope presented on the MHC class I may be important in MS pathogenesis [142].

### 4.2. Effect of Reduced Copy Number of a Proteasome Gene on Disease Susceptibility

Another way by which the proteasome may contribute to disease is by increasing disease related liability in cells, thereby resulting in reduced numbers of diseased cells. A distinct class of cancer-specific liabilities resulting from genome instability was recently reported [145]. Utilization of both genome wide copy number and loss of function data (RNAi profiles) from 86 cancer cell lines identified predominantly proteasome, spliceosome, and ribosome components that were associated with associated with copy-number loss [145]. Cells containing partial *PSMC2* copy-number loss lack a proteasome complex composed of the protein product of *PSMC2*, Rpt1, and three other 19S subunits and eventually die after *PSMC2* suppression [145].

## 5. Polymorphisms in Genes That Code for Proteins Which Directly Interact and Affect Proteasome Function

Besides directly having polymorphisms on proteasome subunits which affect proteasome function, proteasome interacting proteins are also likely to have mutations that affect proteasome function. An E201 deletion in the proteasome 26S ATPase subunit 3-interacting protein (PSMC31P), which is highly expressed in testis and colon, has been associated with XX ovarian dysgenesis [146]. XX ovarian dysgenesis is characterized by primary amenorrhea, lack of spontaneous pubertal development, hypergonadotropic hypogonadism, and uterine hypoplasia as a result of streak gonads [146]. PSMC3IP enhances the meiotic recombination protein DMC1-mediated strand exchange needed for pairing homologous chromosomes during meiosis and has been shown to modulate the activity of proteasomes through association with PSMC3 [146–148]. However, the effect of the E201 deletion on proteasome function has not been determined.

Polymorphisms in other proteins such as the proteasome maturation protein (POMP) are also known to be associated with rare diseases. A one base pair deletion (−95C) in POMP is associated with keratosis linearis, ichthyosis congenital, and sclerosing keratoderma (KLICK syndrome) in several European families [149]. POMP associates with *α* and *β* proteasome intermediates and facilitates the assembly of *β* subunits onto the *α* subunit rings [101, 150]. Investigation of skin biopsies from KLICK syndrome patients showed altered epidermal distribution of POMP, *α*4 (*PSMA7*), and *β*5 (*PSMB5*), when compared to controls [149]. KLICK syndrome is therefore most likely associated with impaired proteasome assembly which would result in altered proteasome activity and function. Measurement of proteasome activity in diseased skin samples is needed to determine if proteasome activity is decreased.

## 6. Problems Associated with Proteasome Activity Measurements and Need for Measurement of All Proteasome Proteolytic Activities

Both where (from what tissue) and how the proteasome activity is measured are important. Proteasome activity measurements in different human tissues require a basic understanding of the numerous nonproteasomal proteases in tissues (some of which can also cleave proteasomal substrates) and the proper use of proteasome-specific inhibitors. While the most commonly used proteasome inhibitor, MG132, is cheap and works well as a proteasome inhibitor for measuring chymotrypsin-like activity of the proteasome, it is not a good inhibitor for measuring the caspase-like or trypsin-like activity of the proteasome. MG132 is known to inhibit other proteases besides the proteasome including calpains [151] and cathepsins A, B, and K [152–154].

The source of the sample is also important, since proteasomes show tissue-dependent differences in composition, interacting partners, and posttranslational modifications, possibly due to differences in protein expression in tissues [155]. All proteasome measurements related to diseased tissues containing proteasome polymorphisms reported so far measured only the chymotrypsin-like activity of the proteasome. Since the proteasome has three main types of proteolytic activity, it is important to measure the caspase-like and trypsin-like activities of the proteasome as these activities all seem to be partly independent of each other [156–158].

## 7. Gene-Environment Interactions

After many years of intensive investigations for genetic risk factors, no single genetic risk factor is used for risk assessment. More recent genome-wide association (GWA) studies further reveal novel genetic factors that contribute to disease risk. However, the replication of many of these GWA studies is still needed. Replication of some GWA studies showed that some populations are more likely to be affected by certain polymorphisms than other populations with the same polymorphism. This is likely due to complex gene-environment interactions. Gene-environment relations are not well understood, but recent evidence suggests that these relationships may be more important than those previously known. Most, if not all, diseases result from complex interactions between an individual's genetic makeup and environmental factors. People with different genetic variations sometimes respond differently to the same environmental exposure. A recent study using pooled data from 24 studies of the Breast Cancer Association Consortium (34,793 invasive breast cancers and 41,099 controls) showed that the risk of breast cancer associated with some common genetic variants varies with environmental risk factors (such as alcohol consumption and parity) [159].

## 8. Conclusions

**Figure 12:**
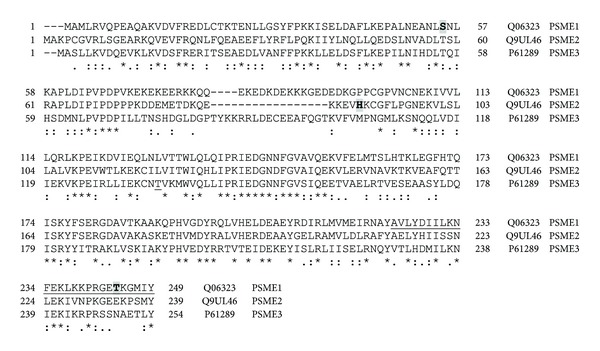
Alignment of human PSME subunits 1–3. Protein sequences of the three proteasome PSME subunits were aligned using Clustal W. ⋆, identical residue in all six subunits;  :, conserved amino acids with strongly similar properties;  ., conservation between residues of weakly similar properties. Naturally occurring variants are highlighted with grey boxes. Alternatively spliced regions are underlined. Amino acid residue numbers are shown on the left and right of each sequence and the UniProt accession number and gene name of each sequence are shown to the right of each sequence.

**Figure 13:**
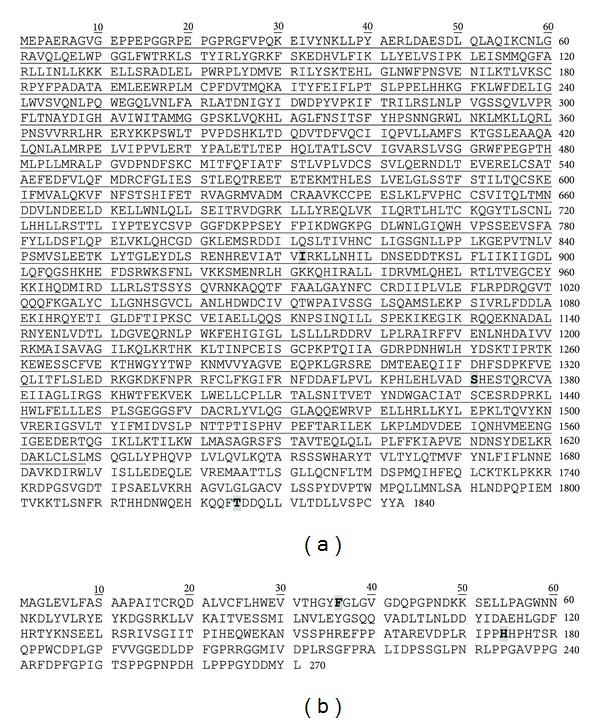
Sequences of human PSME4 and human PSMF1 subunits. Naturally occurring variants are highlighted with grey boxes. Alternatively spliced regions are underlined. Amino acid residue numbers are shown on the right of each sequence. UniProt accession numbers for PSME4 and PSMF1 are Q14997 and Q92530, respectively.

In the last five years, genetic studies have significantly increased our basic understanding of genes associated with diseases. Several disease-associated and promising disease-related candidate genes have been determined for diseases ranging from cardiovascular diseases to immune diseases. The known number of polymorphisms associated with disease and the number of diseases associated with polymorphisms are both likely to rise significantly over the next decade. Numerous mutations and polymorphisms in other proteasome genes (Table 1 and Figures 4, 5, 9, 10, 12, and 13) are already known, but the functional consequences of these genetic variations are not known. Several mutations in proteasome genes not associated with disease have been found in diseased tissues, such as a somatic mutation in Rpt6 (*PSMC5*), R60Q, found in a colorectal cancer sample [39]. Understanding whether or not these proteasome mutations are important in disease development will require basic and advanced research to determine how these mutations affect proteasome function and how they affect the cell's physiology. Another question that still needs to be answered is what factors contribute to some polymorphisms having a strong association with diseases in one or a few ethnic groups but not in other ethnic groups.

Studies involving tissues from patients have also increased our understanding of the pathophysiology of these diseases. While measurement of proteasome activity in diseased tissues is important, measurement of purified proteasome activities in these tissues is also needed to determine if the effects of the polymorphism are directly due to modulation of the proteasome or due to indirect effects. It is possible that an amino acid change in a proteasome subunit may cause altered proteasome activity by affecting its interaction with certain enzymes (e.g., preventing or reducing phosphorylation at certain sites), or by affecting weak associating proteins which alter proteasome activity. Another factor that is not yet considered when determining the role of polymorphisms on proteasome function is the large number of posttranslational modifications that occur on proteasome subunits [27, 160–162]. The heterogeneity of posttranslational modifications on proteasome subunits depends on many factors which will vary significantly between individuals. The most common posttranslational modification is possibly phosphorylation, which can be removed by nonspecific phosphatases, allowing dephosphorylated, purified proteasomes from normal and diseased tissues to be compared. Ideally, expression of wild-type and mutant proteasome subunits which are integrated into the intact 26S proteasome in a cell culture system would allow the researchers to determine if posttranslational modifications are major considerations when defining the role of SNPs in proteasome functions.

Positive associations between a polymorphism and a disease in case control association studies are often not replicated in independent studies, as the design of many studies lack the statistical power to properly detect any potential association [163]. In general, larger population sizes are needed for association studies. When large population studies are unavailable, but enough “smaller” studies are available, meta-analysis of GWA studies should be carried out. Better collaboration between research groups and even countries is needed to allow significantly larger population studies to be conducted. These larger studies are critical to help unravel the effects of environmental factors on disease related polymorphisms. Besides limited sample size, problems due to false-positive results and publication bias are still a significant problem [164].

The current standard of using phenotypic biomarkers for clinical prognosis will continue for the foreseeable future since these biomarkers integrate both genetic and nongenetic factors. Nevertheless, in the near future it is likely that genotyping for specific SNPs will be useful in clinical diagnosis and prognostic assessment of patients. SNP markers are already being used in the diagnosis of a few diseases such as Wilson disease [165]. An understanding of how the gene polymorphisms affect proteins associated with disease will likely lead to new drug targets and therapeutic approaches.

## Figures and Tables

**Table 1:** Names and characteristics of human proteasome genes.

Gene name	Protein name	Other names	Chromosome location	Sequence length	MW (Da)	First methionine removed	Polymorphisms	Reference
20S subunits								
PSMA1	*α*6	C2, Pro-*α*5, *α*6_sc, nu, Pros30, p30k, Pre5, HC2, PSC2	11p15.1	263	29556	No	rs17850016 (G37V)	[36, 37]
PSMA2	*α*2	C3, Pro-*α*2, *α*2_sc, Pre8, Prs4, Y7, HC3, PSC3, Lmpc3	7p14.1	233	25767	Yes	(L110V)	[38, 39]
PSMA3	*α*7	C8, Pro-*α*7, *α*7_sc, Pre10, Prs1, C1, Prc1, HC8, PSC8	14q23	254	28302	Yes		[40]
PSMA4	*α*3	C9, Pro-*α*4, *α*3_sc, Pre9, Prs5, Y13, HC9, PSC9	15q25.1	261	29484	No		[41]
PSMA5	*α*5	Zeta, Pro-*α*1, *α*5_sc, Pip2, Doa5, [Pup2]	1p13	241	26411	No		[42]
PSMA6	*α*1	Iota, Pro-*α*6, *α*1_sc, Pros27, pre27k, C7, Prs2, Y8, Scl1	14q13	246	27399	No	rs1048990 (-8C-G), rs15434 (A233S)	[37, 43, 44]
PSMA7	*α*4	C6, Pro-*α*3, *α*4_sc, XAPC-7, Pre6	20q13.33	248	27887	No	335C-A (A112D)	[38, 45]
PSMA8	—	PSMA7L	18q11.2	256	28530	No		[46]
PSMB1	*β*6	C5, gamma, Psc5, Pre7, Prs3, Pts1	6q27	241	26489	No	rs12717 (P11A), rs10541 (I208N)	[36, 47]
PSMB2	*β*4	C7, Pre1, C11, C7-I, HC7-I	1p34.2	201	22836	No		[48]
PSMB3	*β*3	C10, theta, Pup3, C10-II	17q12	204	22818	Yes	rs4907 (M34L)	[48, 49]
PSMB4	*β*7	N3, beta, Pros26, HsN3, Pre4, Rn3, Lmp3	1q21	264/219*	29204/24392*	No	rs1804241 (M95I), rs4603 (I234T)	[46, 48–51]
PSMB5	*β*5	X, epsilon, LmpX, MB1, Pre2, Doa3, Prg1	14q11.2	263/204*	28480/22458*	No	rs11543947 (R24C)	[44, 48, 52]
PSMB6	*β*1	Y, delta, LmpY, Pre3, Lmp19	17p13	239/205*	25358/21904*	Yes	rs2304974 (P107A)	[44, 52]
PSMB7	*β*2	Z, alpha, Pup1, Mmc14	9q34.11-q34.12	277/234*	29965/25295*	No	rs4574 (V39A)	[36, 53]
PSMB8	*β*5*i*	Lmp7, Psmb5i, Ring10, Y2, C13, Mc13	6p21.3	276/204*	30354/22660*	No	rs114772012 (G8R), (PGH30-32RPD), rs2071543 (Q49K), rs17220206 (T74S), (T75M), (G201V)	[46, 54–57]
PSMB9	*β*1*i*	Lmp2, Psmb6i, Ring12	6p21.3	219/199*	23264/21276*	No	rs35100697 (G9E), rs241419 (V32I), rs17587 (R60H), rs17213861 (R173C)	[58, 59]
PSMB10	*β*2*i*	MECL-1, Lmp10	16q22.1	273/234*	28936/24648*	No		[60]
PSMB11	*β*5*t*	beta5i-like, beta5t	14q11.2	300/251*	32530/27232*	No	rs34457782 (G49S)	[34]

19S proteasome								
PSMC1	Rpt2	S4, Yhs4, Yta5, P26s4	14q32.11	440	49185	Yes		[38]
PSMC2	Rpt1	S7, Mss1, Yta3, Cim5, Nbla10058	7q22.1-q22.3	432	48503	Yes		[61]
PSMC3	Rpt5	S6a, S6′, p50, Tbp1, Yta1, Sata	11p11.2	439	49204	No		[61]
PSMC4	Rpt3	S6b, S6, Mip224, Tbp7, Yta2, Ynt1, Cip21	19q13.11-q13.13	418	47336	No		[61]
PSMC5	Rpt6	S8, p45, Trip1, Sug1, Cim3, Crl3, Tbpy, Tby1	17q23.3	405	45495	Yes	(R60Q), rs11543211 (R258W)	[39, 44]
PSMC6	Rpt4	S10b, p42, Sug2, Prs10, Pcs1, Crl13, CADP44, P44	14q22.1	389	44173	No		[38]
PSMD1	Rpn2	S1, p112, Sen3	2q37.1	953	105836	No		[60]
PSMD2	Rpn1	S2, p97, Trap2, Hrd2, Nas1, Rpd1, Protein 55.11	3q27.1	908	100200	No	rs11545172 (A176T), rs11545169 (E313D), rs17856236 (N724Y)	[44, 61]
PSMD3	Rpn3	S3, p58, Sun2, P91a, Tstap91a	17q21.1	534	60978	No		[60]
PSMD4	Rpn10	S5a, ASF1, Mcb1, Sun1	1q21.3	377	40737	No		[61]
PSMD5	—	S5b, KIAA0072	9q34.11	503	56065	Yes	rs2297575 (E21G), rs17282618 (L72H)	[60, 61]
PSMD6	Rpn7	S10a, SGA-113M, p44S10, p42A, PFAAP4, KIAA0107	3p21.1	389	45531	No		[60]
PSMD7	Rpn8	S12, p40, Mov34L	16q23-q24	324	37025	No		[62]
PSMD8	Rpn12	S14, p31, Nin1	19q13.2	350	39612	No		[60]
PSMD9	—	S15, p27	12q24.31-q24.32	223	24682	No	rs2230681 (V17A), rs2291116 (T74I), rs1177573 (R134W), rs1177573 (E197G)	[50, 63–65]
PSMD10	Gank-yrin	p28, p28(GANK)	Xq22.3	226	24428	No		[38]
PSMD11	Rpn6	S9, p44.5, Nas4	17q11.2	421	47333	Yes		[38]
PSMD12	Rpn5	p55, Nas5	17q24.3	455	52773	Yes	rs2230680 (V358A)	[60]
PSMD13	Rpn9	S11, p40.5, Les1, Nas7	11p15.5	376	42945	No	rs1045288 (N13S), rs28927679 (S150L), rs1794108 (G204E), rs1794109 (L205F)	[36, 66–68]
PSMD14	Rpn11	Poh1, Mpr1, Mad1, Pad1, PAD1 homolog 1,	2q24.2	310	34577	No		[38]

Proteasome activators								
PSME1	PA28*α*	PA28A, IFI5111, 1S REG-alpha	14q11.2	249	28723	No	rs1803830 (S55N), rs14930 (T244K)	[48, 52]
PSME2	PA28*β*	PA28B, 1S REG-beta	14q12	238	27270	Yes	rs7146672 (H89P)	[48, 60, 69]
PSME3	PA28*γ*	PA28G	17q21.31	253	29375	Yes		[60, 70]
PSME4	PA200	KIAA0077, 1S REG-gamma	2p16.2	1843	211334	No	rs2302878 (I872V), rs805408 (S1371T), rs35903236 (T1825A)	[38, 44]

Proteasome inhibitor								
PSMF1	PI31		20p13	271	29817	No	rs1803415 (F36C), rs2235587 (H176R)	[60, 71]

*Mature form of protein after propeptide is removed. When the first residue (methionine) of some proteins is removed, the molecular weights and sequence length given represent the mature forms of the proteasome subunit with the methionine removed.

**Table 2:** Polymorphisms in proteasome genes associated with human diseases.

Gene	Polymorphism	Amino acid change	Disease	References
20S subunits				
PSMA6	−8C>G (rs1048990)	—	Myocardial infarction	[60, 72, 73]
			Type 2 diabetes	[74, 75]
			Ischemic stroke	[72]
			Coronary artery disease	[60]
PSMA7	335C>A	A112D	Intellectual disability	[45]

19S subunits				
PSMD3	SNPs rs4065321 and rs709592	—	Diabetes	[76]
PSMD7	SNP, rs17336700 in intron 3	—	Ankylosing spondylitis	[18]

Immunoproteasome subunits				
PSMB8	c.224C>T	T75M	JMP syndrome	[54]
		G210V	Nakajo-Nishimura syndrome	[57]
	c.224C>T, c.405C>A	T75M	CANDLE syndrome	[77]
		Q145K	*M. tuberculosis* infection	[78]
	LMP-K/Q	—	Cancer	[79]
	LMP-Q/Q	—	Ankylosing spondylitis	[80]
	G/T-37360	—	Type 1 diabetes mellitus	[81]
PSMB9	HLA-B27	—	Graves' disease	[82]
	179G>A	R60H	Ankylosing spondylitis	[83]

Table shows only disease-associated polymorphisms for which the SNP or amino acid change is known.
